# One-pot method of synthesis of Pt/SnO_2_ system and its electrocatalytic activity

**DOI:** 10.1186/s13065-014-0049-0

**Published:** 2014-08-23

**Authors:** Agnieszka Martyla, Maciej Kopczyk, Piotr Marciniak, Robert Przekop

**Affiliations:** Institute of Non-Ferrous Metals Division in Poznan, Central Laboratory of Batteries and Cells, 12 Forteczna St., 61-362 Poznan, Poland; Adam Mickiewicz University Foundation Poznan Science and Technology Park, 46 Rubiez St., 61-612 Poznan, Poland; Centre of Advanced Technologies Adam Mickiewicz University, 6 Grunwaldzka St., 60-780 Poznan, Poland

**Keywords:** Sol–gel, Electrocatalyst, Low temperature treatment, One-pot method

## Abstract

**Background:**

Electro-oxidation of methanol in acidic solution was investigated on a Pt/SnO_2_ based electrocatalyst obtained by the sol–gel method. Pt/SnO_2_ systems were prepared by *one pot* synthesis using a sol–gel method and tin (IV) acetate as a precursor of SnO_2_ and water solution of hexachloroplatinum acid as a source of metallic phase.

**Results:**

The described method, thanks to its simplicity and mildprocessing temperature, offers uniform dispersion of metal phase in the bulk of the gel forming as a result of hydrolysis and condnastionon of tin precursor. It has been found that the obtained system exhibits high electrocatalytic activity just after low temperature processing. This work investigates the effect of the Pt concentration and the influence thermal treatment of Pt/SnO_2_ on its electrochemical activity.

**Conclusions:**

The described procedure is a simple method of producing a highly active Pt/SnO_2_ electrocatalyst without using thermal treatment in reducing conditions. The oxidation state of Pt is a determining factor for its activity in an electrooxidation process.

**Graphical Abstract:**
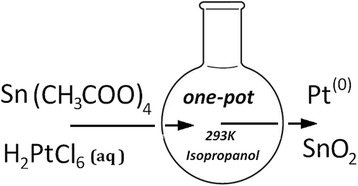
One-pot method of synthesis of Pt/SnO_2_ catalyst

## Introduction

Platinum is the most critical single-component catalyst used in taking place in low temperature fuel cells because of its catalytic activity in electrooxidization reactions performed at room temperature. It is one of few metal [1,2] that can withstand acidic conditions, inside a fuel cell, but it is also very expensive [3] and can be readily poisoned by carbon monoxide, an intermediate in the methanol electro-oxidation reaction. In order to improve the methanol oxidation kinetics, platinum is usually alloyed with another metal such as ruthenium, which is more effective in dissociating water [4] or supported on metal oxides. Protons transfer to the metal oxide support surface can create clean active sites on the surface of platinum and prolong the effective dehydrogenation activity of the catalyst. Metal oxides are also stable under operating conditions of fuel cells and are resistant to oxidation [3]. Tin oxide was reported to be able to enhance the activity of the platinum catalyst for methanol and ethanol electrooxidation. Doped SnO_2_ is one of the most promising catalyst support material for fuel cells due to its chemical stability and high promoting effect [5-7]. The lattice structure of the tin (IV) oxide substrate allows for easy manipulation of pore size, making it a favourable material. This is important since the substrate is impregnated with nanosized platinum particles. Being able to control the pore size distribution can help make the process more effective by even platinum distribution over the entire surface with a desired concentration [8]. For these reasons, searching for new, simple and effective synthesis methods of highly active electrocatalysts is still a challenge. *One–pot reaction* synthesis method is well known in organic chemistry as a strategy of improving efficiency and selectivity of chemical processes by conducting them in one reaction set with no isolation of intermediates [9,10]. In the field of catalytic materials synthesis, this term is not so popular, although, methods of obtaining complex systems sometimes may be simplified in a similar manner and lead to increase in productivity [10].

In this study, Pt/SnO_2_ catalysts with different metal content: 4.1, 8.2 and 16.4% of Pt were obtained by one-pot sol–gel method. Tin (IV) acetate as a gel precursor of tin (IV) oxide and aqueous solution of H_2_PtCl_6_ as a precursor of the metallic phase were applied. An aqueous solution of hexachloroplatinic acid added to isopropyl alcohol solution of tin (IV) acetate causes rapid cross-linking of colloidal SnO_2_. Pt/SnO_2_ sol–gel system have found various applications in catalysis, gas sensors and electrochemistry [11-15]. The sol–gel technique allows to obtain mesoporous oxide as a result of medium temperature treatment (473–573 K) of the systems and consequently may positively affect the stabilisation of small particles of metallic phase [16].

Platinum crystallite size plays a key role in catalysis and electrochemistry [17]. It is equally important to ensure their stability and maintain their size during the whole operating time of the catalyst. In our method, uniform dispersion of the metal phase precursor in the bulk of the gel occurs during its synthesis, resulting in further metal stabilisation during thermal treatment.

The aim of the study was to determine the effect of the Pt concentration in the media and the effect of the thermal treatment in the temperature range of 293 K to 773 K on the metal crystallite size on the surface of catalyst. The resulting systems were characterized by XRD studies and transmission electron microscopy. The drying and thermal treatment of the Pt/SnO_2_ gels were characterized by ATR/FT-IR spectroscopy and thermal gravimetric analysis. Finally, the electrochemical activity of Pt/SnO_2_ systems was studied by cyclic voltammetry.

## Results and discussion

**Figure 1:**
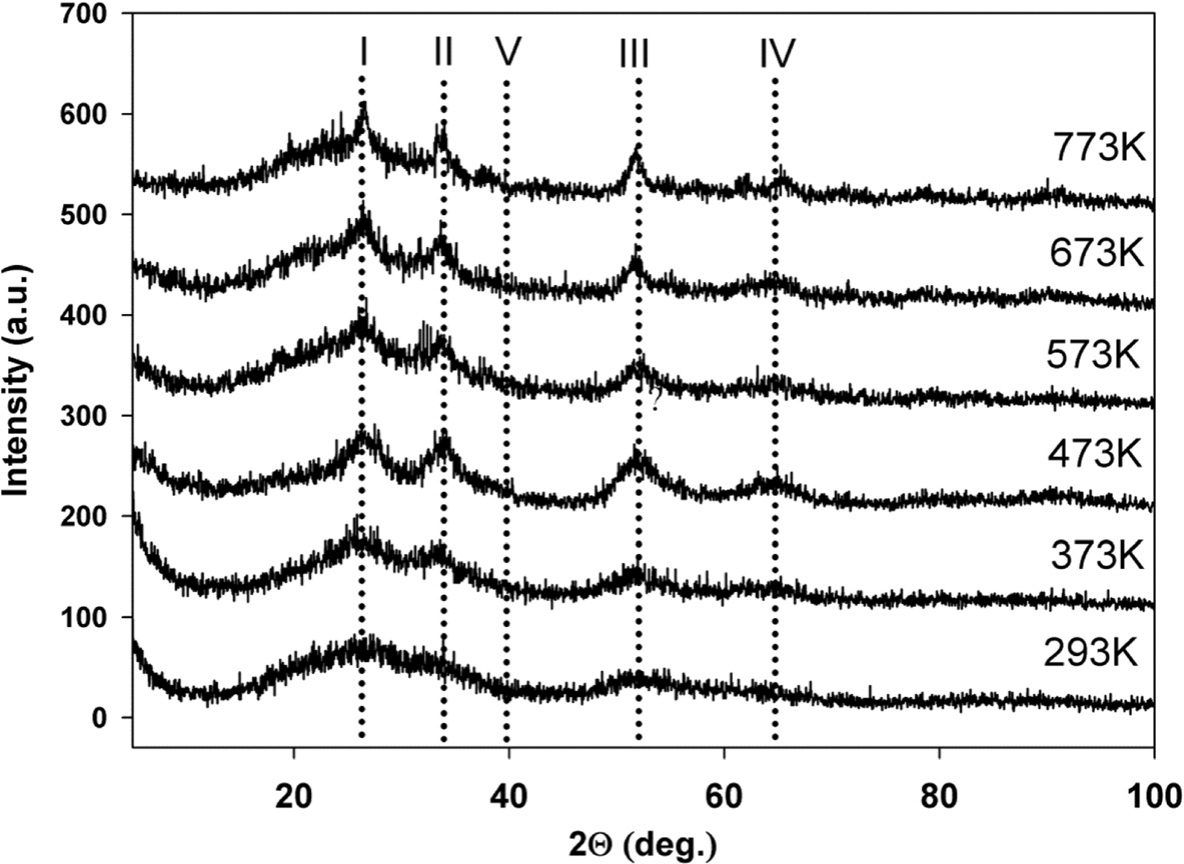
**XRD patterns of 4.1% Pt/SnO**_**2**_ **system.**

**Figure 2:**
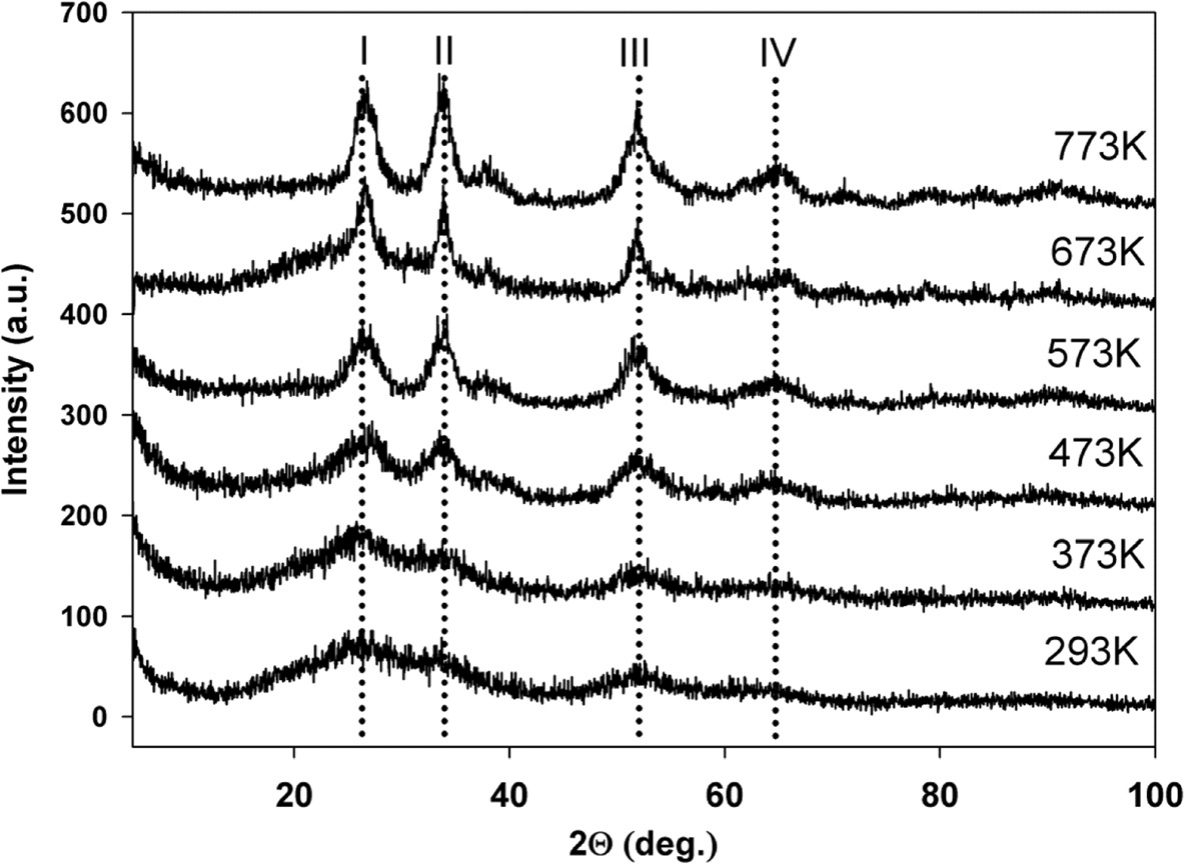
**XRD patterns of 8.2% Pt/SnO**_**2**_ **system.**

**Figure 3:**
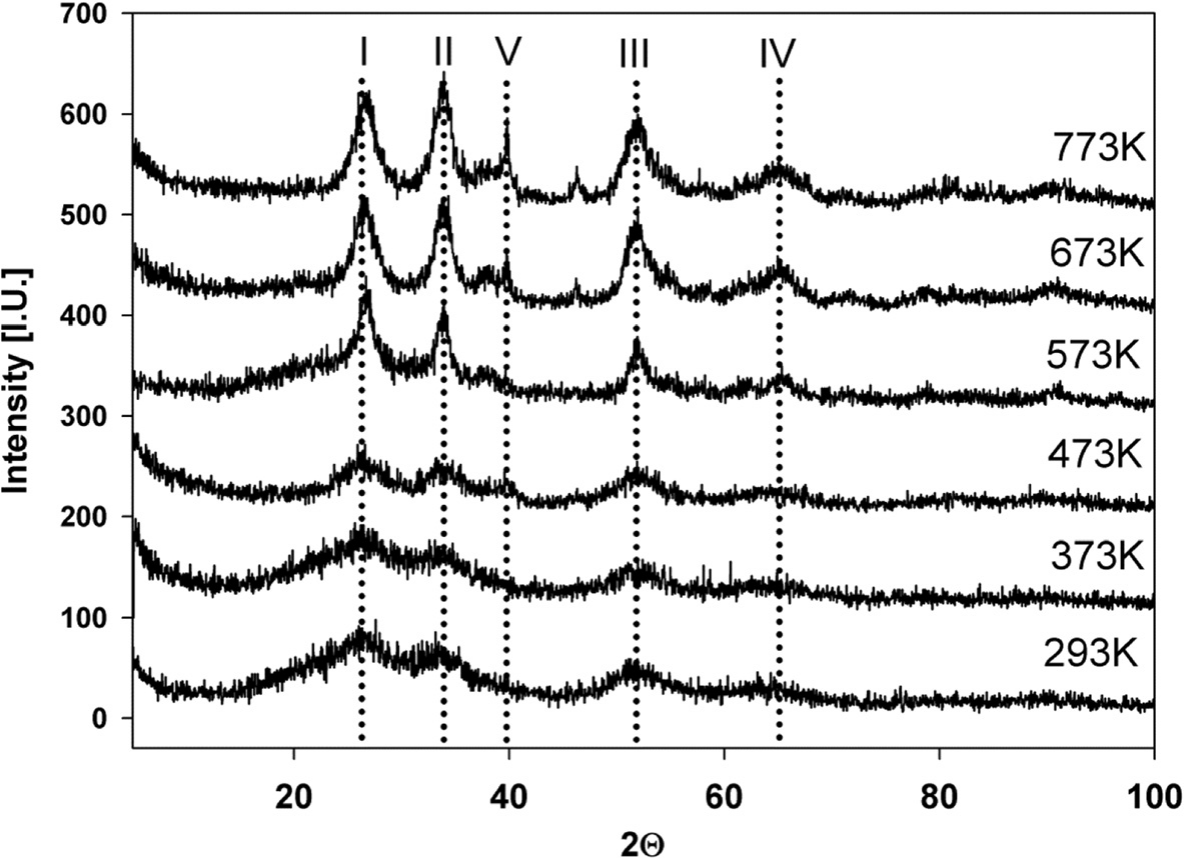
**XRD patterns of 16.4% Pt/SnO**_**2**_ **system.**

X-ray diffraction analysis was performed to study the effect of thermal treatment temperature and metal phase the concentration on the electroactivity. XRD pattern of the as-synthesized systems is typical for all gel products after air-drying (Figures 1, 2 and 3) and shows predominantly only amorphous structure. The XRD diffractogram measured for Pt/SnO_2_ samples presents reflections becoming more defined from the tetragonal crystallographic phase (cassiterite) of SnO_2_ after heating (temperatures from 373 K to 773 K). For higher temperatures, the diffraction peaks become progressively more intense and sharp. Miller indexes are indicated on each diffraction peak. The reflection peaks at ~26 (2theta)/{110}, ~33 (2theta)/{101} at ~51 (2theta)/{211}, at ~65 (2theta)/{301}, can be readily indexed to a tetragonal rutile structure of SnO_2_ (PDF 4+ Card File No. 04-003-5853).

Generally, for a given composition of the system, an increase in the average mean SnO_2_ crystallite size with the increasing the thermal treatment temperature is observed. The samples dried at room temperature and heated at 373 K and 473 K are also amorphous. Furthermore, for all systems and for each Miller index, the SnO_2_ crystallite size is the biggest after thermal treatment at 773 K. The higher load of platinum decreases the intensity of XRD reflexes in comparison to systems with lower metal content (Figures 2, 3). The cassiterite structure is not influenced by the addition of platinum except the 16.4% Pt on SnO_2_ system (Figure 3). It is believed that for other systems it is related to the small size of the platinum crystallites which cannot be detected [18].

Despite a high load of metal phase (up to 16%) no reflexes of platinum crystals or its compounds can be observed. This is probably a confirmation of high dispersion and stabilization of platinum in the tin dioxide gel structure.

**Figure 4:**
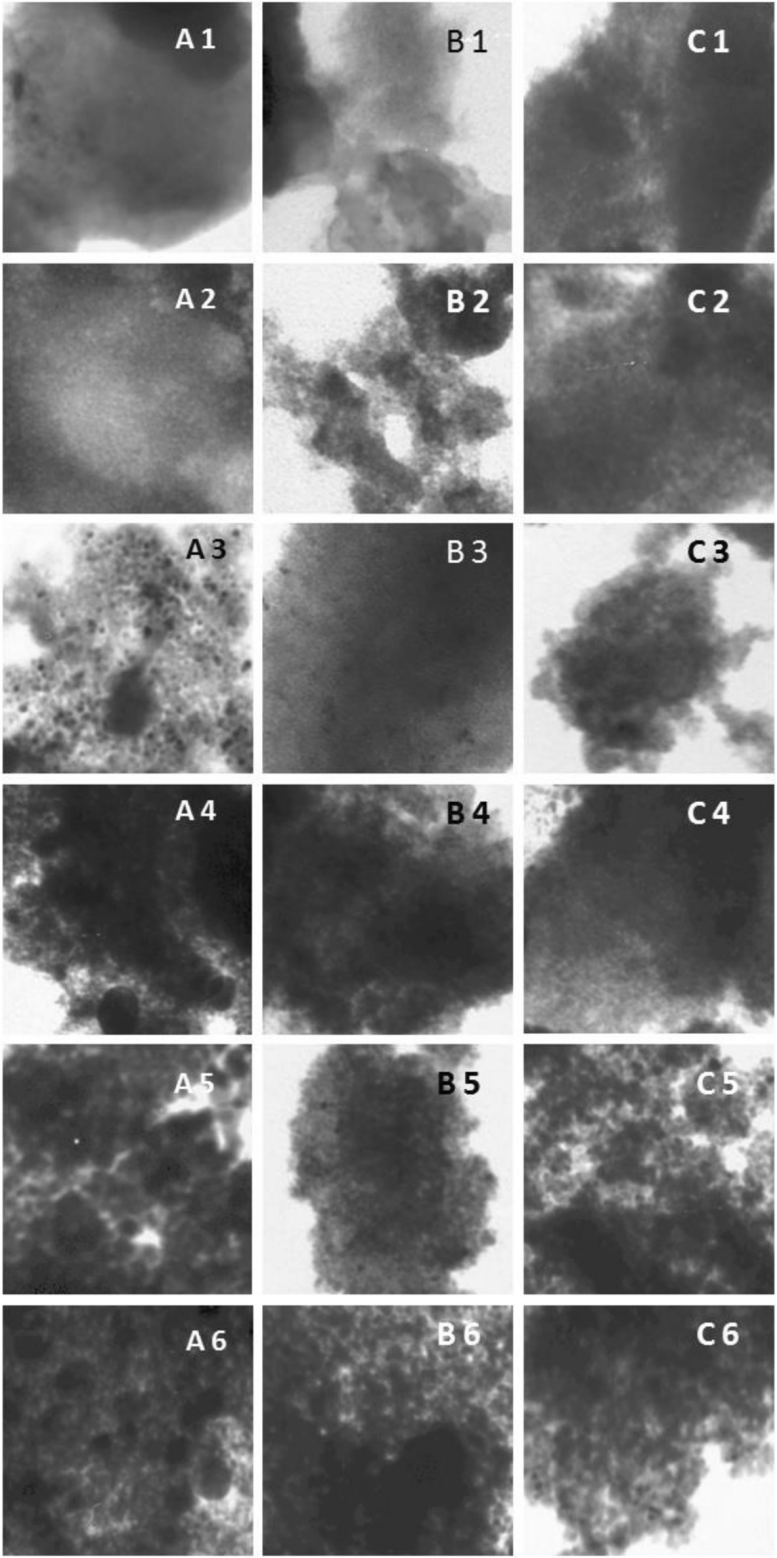
**TEM images of Pt/SnO2 systems, A= 4.1% Pt, B= 8.2% Pt, C= 16.4% Pt.** A1-A6 – **A** system after thermal treatment. B1-B6 – **B** system after thermal treatment. C1-C6 – **C** system after thermal treatment. Temperatures of thermal treatment: 1-293 K, 2 – 373 K, 3 – 473 K, 4 – 573 K, 5 – 673 K, 6 – 773 K.

TEM observations are presented in Figure 4. Images show the area of 200 × 200 nm. Changes in the material and well-defined spherical nanoparticles of Pt which are within the inner mesoporosity of the sol–gel SnO_2_ oxide [19-22]. It seems that the porous structure of SnO_2_ in the obtained system can take part in stabilization of small Pt clusters. Effect of stabilization of small metallic clusters has been described in papers [16]. By the described one-pot reaction method, one can obtain significantly smaller size Pt crystallites than by other synthesis methods of similar materials [7,23].

As it can be seen in Figures 4 A3-A6, B3-B6, C3-C6, the Pt particles agglomerate extensively. Though the primary particle size is below 2 nm, they aggregate to larger particles with an average size near 20–30 nm (Figure 4A6) and further collide with each other to form conglomerate. The changes can be the best visible for the sample with the lowest platinum content. Platinum crystallites coalescence is accompanied by the sintering process, but the migration of small metal species can occur even below the temperature of sintering onset (<673 K) [24]. The formation of this structure was clearly explained by the separation of the tin oxide or sub-oxide phase from the inter-metallic compound particles under a mild oxidation atmosphere [25].

For systems with higher metal phase content, changes are milder (B, C), which is also reflected in the XRD patterns. For these systems, the crystalline phase is not visible as Pt diffraction patterns, indicating the presence of small metal crystallites. It can be concluded from microscopic observation that their sizes are in range of 1 to 10 nm, for treatment at temperatures up to 673 K. This result can explain the broad area observed in XRD spectrum for Pt/SnO_2_ systems, indicating the presence of dispersed nanoparticles beside to crystals, clearly indexed by planes. By defining sol–gel synthesis reaction conditions, it is possible to control well Pt/SnO_2_ particle growth within the method.

**Figure 5:**
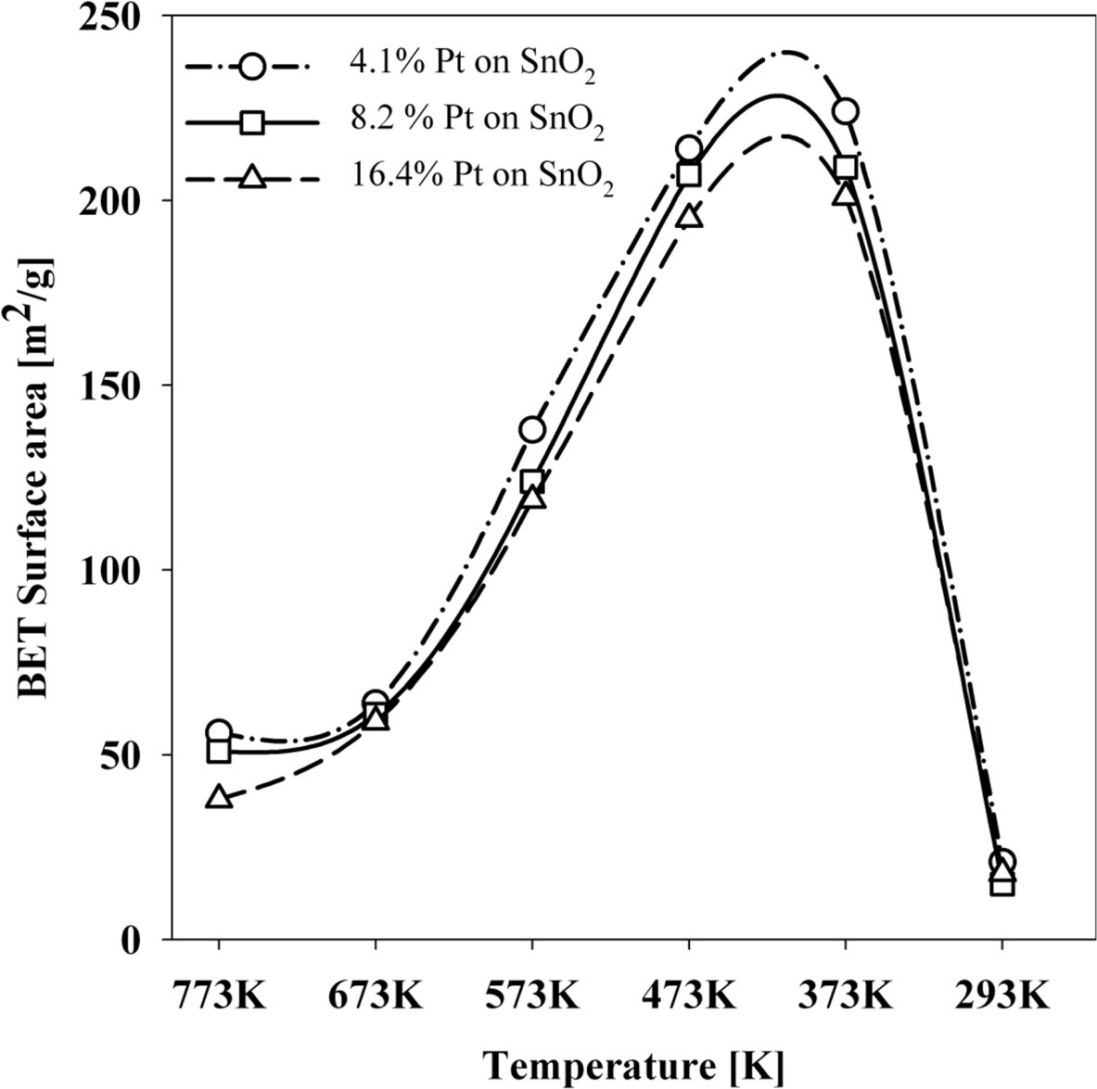
**Surface area of Pt/SnO**_**2**_ **systems.**

The thermal treatment at 373 K results in a rapid increase in surface area which is characteristic for most gel systems obtained by a sol–gel method [26]. For high-temperature treatment we observe a gradual decrease in surface area, up to about 47–50 m^2^/g (after treatment at 773 K). It should be noted that the specific surface area values are significantly higher than for the other porous tin (IV) oxides [27]. We observe the correlation of electrochemical activity and changes in surface area (Figure 5). The decrease in activity is preceded by a decrease in specific surface area and it is associated with the sintering process of small metal particles [28] and with the sintering of nanoparticles - SnO_2_ [29].

**Figure 6:**
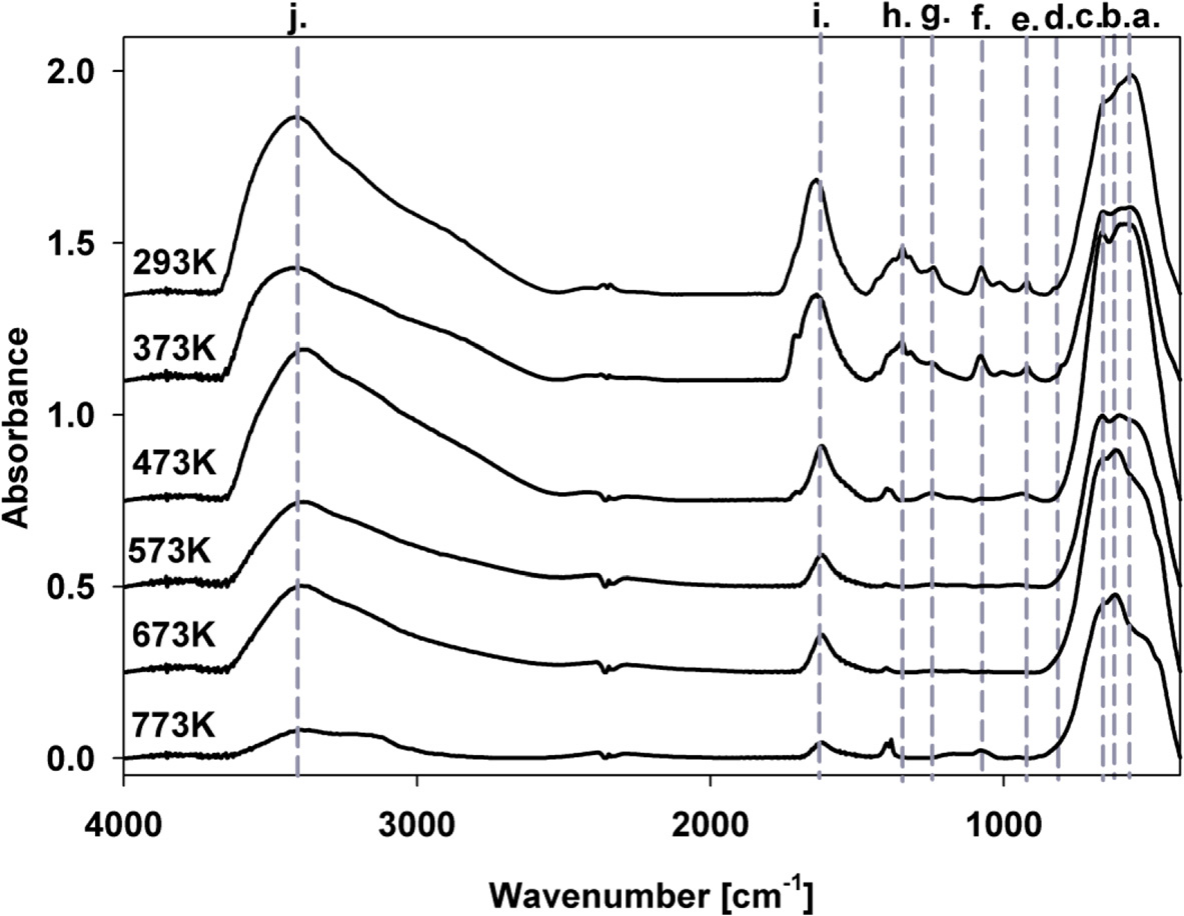
**ATR/FT-IR spectra of 16.4% Pt on SnO**_**2**_ **powder.**

ATR/FTIR spectra of all systems yielded a complex picture – Figure 6. There are no significant differences among spectra of different composition samples for respective temperatures (we present only spectra for 16.4% Pt). Only changes in bands intensity form Pt species can be observed, but in general they are very similar for varying Pt content. Powders show broad bands between 500 and 772 cm^−1^ (Figure 6a,b), which are due to Sn–OH bond stretching. The peak in the region of 1350 cm^−1^ (h) is a complex band from the residual C-H groups and from hexachloroplatinic acid. The H–O–H in-plane deformation is observed at 1634 cm^−1^ (i). In the region of 1700 cm^−1^ (j), we observe a peak associated with the presence of acetic acid [16]. A broad band at 3400 cm^−1^ (k) is attributed to the O–H stretching vibrations from residual alcohol, water, and SnO–H bonds.

ATR/FTIR analysis subsequent to thermal treatment showed an evolution of the absorption bands. Heating eliminates organics residues, because the peaks at 3400 cm^−1^ appear as a shoulder superimposed on the O–H band. They disappear above 673 K - peak at 1700 cm^−1^ (j). The absorption band from 500 to 772 cm^−1^ decreases with the change in intensity of its components (a, b). The SnO_2_ modes can be seen, with the Sn-O-Sn mode at 636 cm^−1^, while a lattice mode of SnO_2_ appears at 772 cm^−1^.

**Figure 7:**
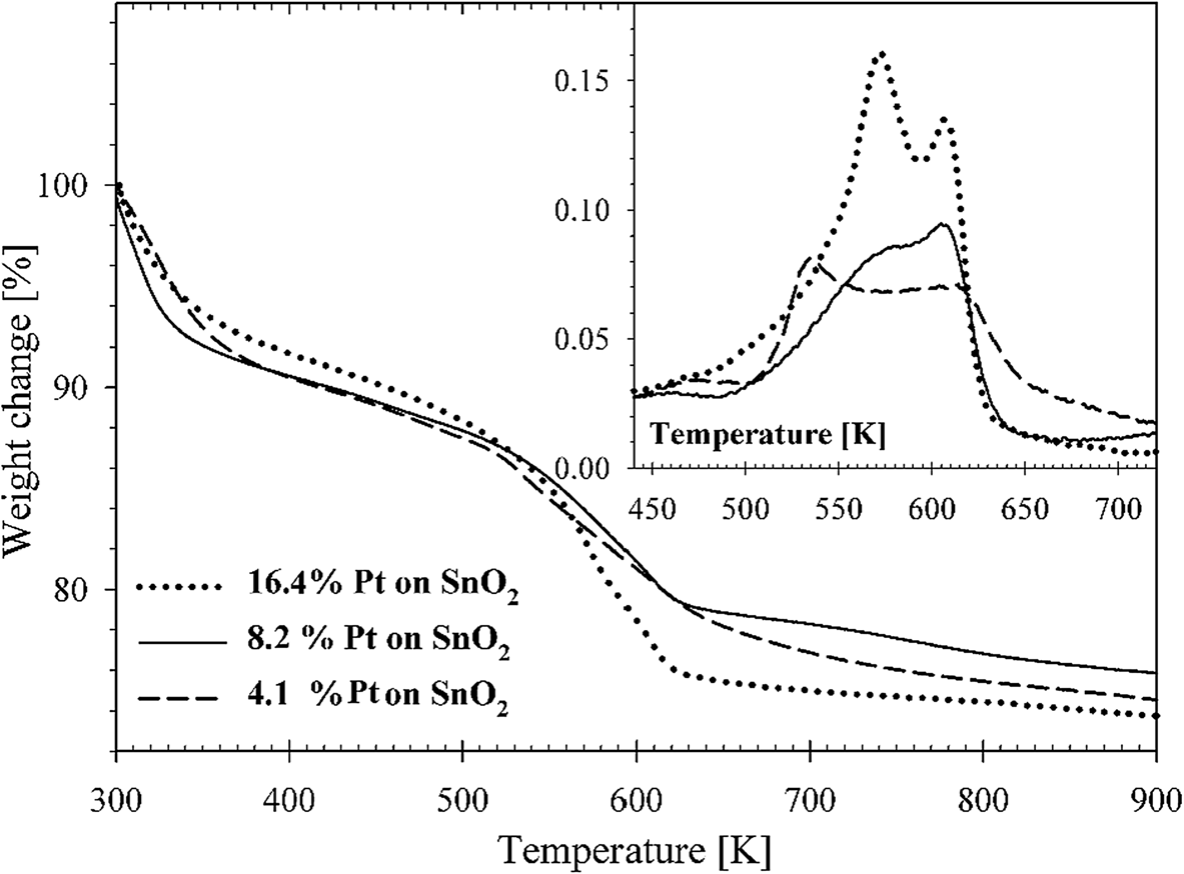
**DTA/TG curves measured on Pt/SnO**_**2**_ **powders.**

Thermogravimetric analysis confirmed the complexity of the thermal decomposition of the starting gel in oxidizing atmosphere. Decomposition occurred in three steps (Figure 7). The first change is observed at 330 K and the corresponding mass loss, evidenced by the TG curve, can be attributed to the desorption of physically adsorbed water and a certain amount of organic solvent. Further effects observed at 525 K and 610 K are attributed to the combustion of the organic residue in the powder [30]. This peak is accompanied by further mass loss, evidenced by the DTG curve. The effect of the organic residues removal is consistent with previously described spectroscopic observations. The change at 525 K can also be affected by decomposition of H_2_PtCl_6_ [31]. Indeed, we observe a linear temperature dependence between the content of the metallic phase and the initial temperature of decomposition process.

**Figure 8:**
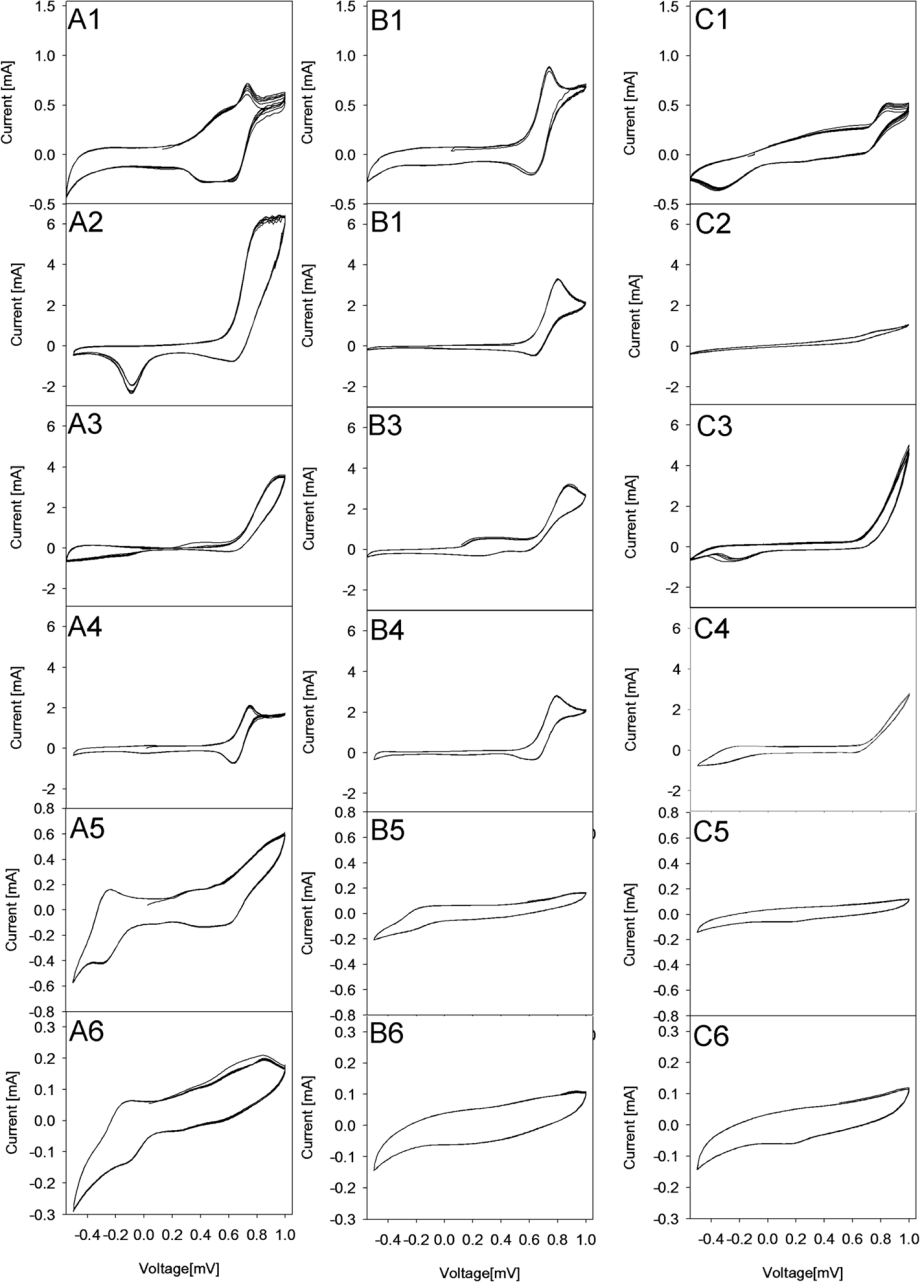
**Cyclic voltammetry of the Pt/SnO**_**2**_ **systems.** A= 4.1% Pt, B= 8.2% Pt, C= 16.4% Pt. A1-A6 – **A** system after thermal treatment. B1-B6 – **B** system after thermal treatment. C1-C6 – **C** system after thermal treatment. Temperatures of thermal treatment: 1-293 K, 2 – 373 K, 3 – 473 K, 4 – 573 K, 5 – 673 K, 6 – 773 K.

The electroactivity of the electrodes towards the oxidation of methanol were investigated by cyclic voltammetry. Voltammetric curves were set so as to sort the electrochemical activity depending on the concentration of the respective systems and the preparation of the platinum catalyst. Figure 8 shows the voltammograms of the unannealed and annealed Pt/SnO_2_ nanocatalysts in 1.0 mol/dm^3^ H_2_SO_4_ with 0.5 mol/dm^3^ CH_3_OH solution.

Each experimental curve of methanol electro-oxidation reaction (Figure 8) shows a peak (in the range of 0.6-0.8 V) which is referred to the direct route of methanol oxidation [32]. The position of the methanol oxidation peak is slightly shifted towards higher voltage values. This phenomenon is observed for 4.1% Pt samples prepared to heating at 473 K and can be related to the formation of Pt agglomerates. For 8.2% Pt systems, the peak is also shifted, for 16.4% Pt systems, the characteristic peak of methanol oxidation is present only for samples treated at room temperature. Treatment at higher temperatures just increases the current.

With increasing the processing temperature, the system activity decreases because of Pt sintering. Larger agglomerates of Pt have lower activity in the methanol oxidation reaction. A significant decline in activity is observed for the systems treated at above 473 K. For the samples treated at temperatures above 573 K, a significant decrease in the activity associated with the sintering of both the metallic phase and the emergence of the carrier porosity is observed, resulting in the CV curve shape changes. Figure 8A5, A6, B5, B6, C5, C6.

The methanol oxidation is a surface sensitive reaction [33]. The shape of the voltammetric curves allows to draw conclusions with respect to platinum crystal faces involved in the reaction. On the basis of literature data [33], one can predict that for most of the systems, the {110} plane – Figure 8A3 can be the predominant crystallographic plane in the surface Pt nanoparticles, but the plane {100} also exhibits an activity – Figure 8B1. The plane {110}, according to the literature data, is the most significant plane favouring the methanol oxidation reaction at an earlier potential and generates most of methanol to CO oxidation current. Though these three electrode systems have reached the same apparent geometric area, the Pt/SnO_2_ systems with 4.1% Pt have much higher current density than other electrodes prepared under the same conditions (373 K). This temperature is the most advantageous from the viewpoint of catalytic activity. It gives the highest current, and the reaction probably proceeds with the greatest speed. This suggests that the structure of the 4.1% Pt/SnO_2_ catalyst can greatly enhance the electrocatalytic activity towards methanol. In this sample Pt nanoparticles uniformly spread in the bulk of SnO_2_, functioning as a multidimensional catalyst. Definitely, the worst results were obtained for the system with the highest content of platinum. In our previous work it was observed that when metal component content on the metal-oxide support increased, the degree of its dispersion and also its activity in the process decreased [34,35].

Curves presented in the Figure 8 clearly show that obtained electrocatalysts are very diverse regarding the properties of their active centres and therefore, their electrochemical activity. Thermal treatment of systems with supported hexachloroplatinic acid has a deep influence on the final form of the metal (its oxidation state). The various nature of redox processes manifesting in different shapes of voltammetric curves which makes them difficult to compare. To simplify that, a ΔI parameter was introduced. Its value is equal to the amplitude of oxidation and reduction current (Eq. 1):

1 $$ \Delta \mathrm{I}=\mathrm{Iox}+\left|\mathrm{Ired}\right| $$

**Figure 9:**
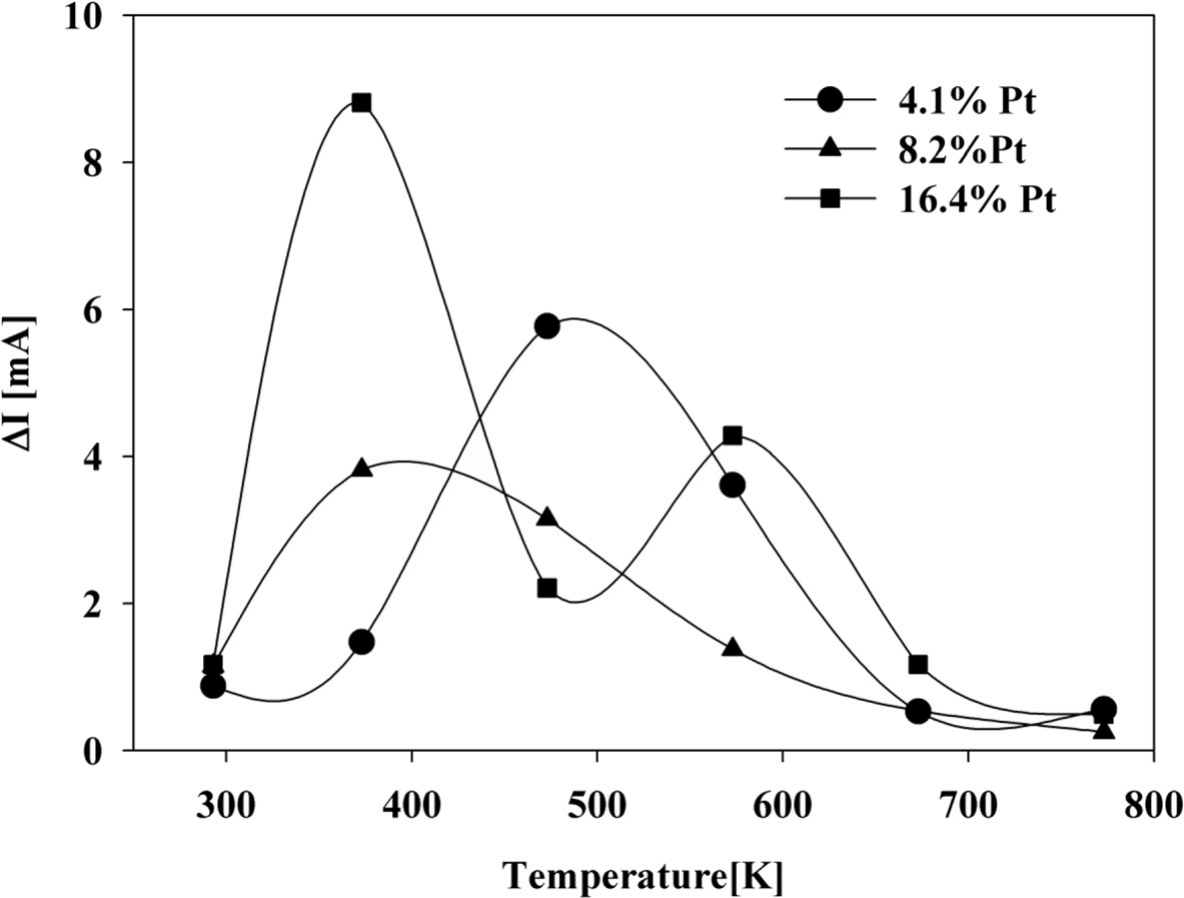
**The sum of oxidation current and absolute value of reduction current as a function of temperature.**

Plots (Figure 9) present values of ΔI of all obtained systems as a function of temperature. Matching these values with a predominant chemical form of platinum at a given temperature that results from subsequent stages of the metal precursor decomposition [36] allows to one draw conclusion regarding its most active form in electrochemical processes. This studies lead to conclusion that hydrated hexachloroplatinic acid (H_2_PtCl_6_ · 6H_2_O) is not active in electrochemical processes. The activity of all system increases rapidly after removal of the coordinating water (Eq. 2). It is the most prominent for high platinum content systems. Plots and collected data show that oxidation state of Pt is a determining factor of its activity and for systems in which platinum is at II or IV oxidation state [32].

2 $$ {H}_2 PtC{l}_6\times 6{H}_2 O\overset{\approx 373 K}{\to }{H}_2 PtC{l}_6\overset{\approx 433 K}{\to } P tC{l}_4\overset{\approx 573 K}{\to } P tC{l}_2\overset{\approx 673 K}{\to } P{t}^{(0)} $$

## Conclusions

Pt/SnO_2_ nanoparticles were synthesized by one-pot sol–gel technique. The proposed method of synthesis and thermal treatment allow for good control of the size of metal and stabilization of nanoparticles within the pores of the support. It was possible to obtain uniform and well dispersed platinum nanoparticles, which was confirmed by XRD and TEM. After thermal treatment, Pt particle migration and coalescence on the surface was observed.

However, this process required higher temperatures in comparison to multi-stage synthesis methods thanks to stabilisation of small Pt clusters in the gel structure. Pt/SnO_2_ - based electrocatalyst showed electrochemical activity during methanol oxidation reaction in acid solution. The CV results demonstrated that the oxidation state of Pt is a determining factor of its activity.

## Experimental

### Preparation Pt/SnO_2_ by the sol–gel technique

The precursor of the tin(IV) oxide was tin (IV) acetate. To 48 cm^3^ of isopropanol, and 8 cm^3^of methanol 1.6 g of the tin (IV) acetate was added and immersed in an ultrasonic bath at 323 K until tin (IV) acetate dissolved. Increasing amounts of Н_2_PtCl_6_ · 6H_2_O solution were added to the tin acetate alcohol solution: 1.35 cm^3^, 2.7 cm^3^ and 5.4 cm^3^ respectively, corresponding to 28.12 mg, 56.24 mg and 112.55 mg of Pt to obtain samples containing 4.1 wt % (series A), 8.2 wt % (series B) and 16.4 wt % (series C) of Pt. After Н_2_PtCl_6_ · 6H_2_O dosing, the systems were dispersed in an ultrasonic bath at 323 K for 12 hours.

### Physicochemical characteristics

The phase identification and the influence of the thermal treatment on SnO_2_ phase was performed using an X-ray diffraction (XRD) powder diffractometer (Philips, PW 1050) using CuKα lamp radiation and Ni filter. X-ray spectra were recorded in the angular range of 5–80 [2theta]. Imaging the surface was performed with SEM electron microscopy (Zeiss EVO 40) and TEM (JOEL JEM 1200 EX).

The thermal analysis of SnO_2_ gel thermal analysis was performed using a TA Instruments gravimetric analyser (TA50) under nitrogen atmosphere, 20 cm^3^/min, at heat rate of 20 K/min and under air atmosphere 20 cm^3^/min, at heat rate of 20 K/min.

Surface studies was carried out using an FT-IR spectrometer (Bruker, TENSOR 27) from ATR accessory (SPECAC). Measurement resolution was 4 cm^−1^.

The cyclic voltammetry (CV) experiment was performed in a two-electrode cell of Swagelok type with the application of ECLAB V10.12 VMP model 0.3 potentiostat/galvanostat by Bio-Logic, in the range of potential from −0.5 to 1.0 V. The scan rate of 0.05 mVs^−1^ was applied.

The catalyst powders were suspended in solution of PVdF-HFP in acetone with graphite and applied onto a stainless steel electrode. The cell’s configuration was: steel-Pt/SnO_2_ | separator | steel. The electrolyte contained 1 mol/dm^3^ H_2_SO_4_ and 0.5 mol/dm^3^ CH_3_OH.
